# Neutrophil-to-lymphocyte ratio as a predictor of post-ablation recurrence in hypertensive patients with paroxysmal atrial fibrillation

**DOI:** 10.7150/ijms.118572

**Published:** 2026-01-01

**Authors:** Zixi Zhang, Chao Sun, Siyuan Tan, Yichao Xiao, Tao Tu, Qiuzhen Lin, Chan Liu, Shunyi Li, Chaoshuo Liu, Cancan Wang, Murong Xie, Qiming Liu

**Affiliations:** 1 Department of Cardiology, The Second Xiangya Hospital, Central South University, Changsha City, Hunan Province, People's Republic of China.; 2 Department of International Medicine, The Second Xiangya Hospital, Central South University, Changsha City, Hunan Province, People's Republic of China.; 3 Department of Orthopedics, Hospital of Traditional Chinese Medicine Affiliated to Xinjiang Medical University, Urumqi, Xinjiang Province, People's Republic of China; 4 The First Detention Area, Central Hospital of Hunan Provincial Prison Administration, Changsha City, Hunan Province, People's Republic of China.

**Keywords:** neutrophil-to-lymphocyte ratio (NLR), atrial fibrillation, hypertension, catheter ablation, recurrence

## Abstract

**Background:** Recurrence of atrial fibrillation (AF) after catheter ablation remains a major clinical challenge in hypertensive patients with paroxysmal AF, and reliable inflammatory predictors for recurrence are lacking.

**Objective:** To assess the predictive value of neutrophil-to-lymphocyte ratio (NLR) for AF recurrence.

**Methods:** Cox regression models, restricted cubic splines (RCS), receiver operating characteristic (ROC) curves, interaction and joint analyses, and mediation analysis were employed to evaluate the relationship between NLR and AF recurrence.

**Results:** AF recurrence occurred in 17.94% of patients. The NLR was independently associated with recurrence (HR: 1.30, 95% CI: 1.12-1.50; *P* < 0.001). RCS analysis revealed a non-linear relationship with a threshold of 2.37, above which the recurrence risk significantly increased. ROC analysis demonstrated stable predictive performance of the NLR. No significant interaction effect between the NLR and blood pressure control status was observed. Regardless of blood pressure control, an NLR ≥ 2.37 was associated with an increased recurrence risk, with the highest risk in patients with both a high NLR and uncontrolled hypertension (HR: 3.92, 95% CI: 1.82-8.42; *P* < 0.001). Sensitivity analysis confirmed the robustness of the findings, and mediation analysis revealed no significant mediating effect.

**Conclusions:** The NLR is an independent predictor of post-ablation recurrence in hypertensive patients with paroxysmal AF. The combination of NLR ≥ 2.37 and uncontrolled hypertension identifies a high-risk subgroup, underscoring the need for integrated anti-inflammatory and blood pressure control strategies. Further prospective studies are needed to validate these findings and evaluate targeted anti-inflammatory strategies.

## 1. Introduction

Atrial fibrillation (AF) is the most prevalent sustained arrhythmia and is frequently observed in individuals with hypertension 1. Hypertension is a well-established risk factor for AF; it contributes to both the development and progression of AF, thereby increasing the risk of major adverse cardiovascular events such as stroke, heart failure, and mortality 2. Approximately 70% of patients with AF have concomitant hypertension, and individuals with hypertension have a 50% higher risk of developing AF compared to those with normal blood pressure (BP) 3. In this high-risk population, early rhythm control is crucial, and catheter ablation has become a cornerstone therapy for drug-refractory AF 4. However, despite its clinical efficacy, AF recurrence after ablation remains a persistent challenge.

Although multiple predictors of AF recurrence have been proposed, their discriminatory power is limited. Traditional indicators—such as left atrial size and comorbidities—provide only moderate prognostic value. Increasing evidence highlights the important role of inflammation in the initiation and maintenance of AF 5, 6. Systemic inflammatory markers, including C-reactive protein (CRP) and interleukin-6, have been linked to AF burden and poor outcomes after ablation 7. However, these markers have limited clinical utility due to variability, cost, and weak predictive performance. The neutrophil-to-lymphocyte ratio (NLR), a readily available and inexpensive biomarker derived from routine blood counts, has emerged as a promising indicator of systemic inflammation and immune dysregulation 8, 9. Elevated NLR has been associated with various adverse cardiovascular outcomes 10-12. Nevertheless, the prognostic value of NLR for predicting post-ablation AF recurrence, particularly in hypertensive patients, remains inadequately explored.

This study aimed to investigate the association between preprocedural NLR and AF recurrence after catheter ablation in hypertensive patients. By evaluating this accessible inflammatory marker, we sought to clarify its potential prognostic value in this vulnerable population.

## 2. Methods

### 2.1 Study population

This observational, retrospective cohort study was conducted in the Department of Cardiology at the Second Xiangya Hospital. A total of 906 adult patients (age > 18 years) diagnosed with AF who underwent catheter ablation—including either radiofrequency ablation (RFA) or cryoballoon ablation (CBA)—between January 1, 2019, and January 1, 2023, were initially screened for eligibility. Patients were excluded if they met any of the following criteria: non-paroxysmal AF (n = 188); absence of hypertension (n = 179); history of repeat ablation (n = 43); missing essential data (n = 4); prior renal denervation (n = 0); acute heart failure (n = 5); severe valvular heart disease (n = 8); acute myocardial infarction (n = 2); significant hepatic or renal dysfunction (n = 6); primary hyperaldosteronism (n = 2); Cushing's syndrome (n = 1); pheochromocytoma (n = 1); active malignancies (n = 3); active infections or autoimmune diseases (n = 0); or pregnancy (n = 0). After applying these exclusion criteria, the final study population consisted of 418 patients. A detailed flowchart of patient selection is provided in Supplementary Figure 1.

### 2.2 Assessment of the NLR

The NLR was calculated using the following formula ^13:^



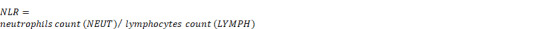



Patients were stratified into quartiles (Q1-Q4) based on baseline NLR values, with Q1 serving as the reference group for comparative analyses.

### 2.3 Data collection

Demographic information, clinical characteristics, comorbid conditions, and laboratory results were extracted from the hospital's electronic medical record system. BP was measured according to standardized protocols. Hypertension was defined as either a mean systolic blood pressure (SBP) ≥ 140 mmHg, mean diastolic blood pressure (DBP) ≥ 90 mmHg, or the use of antihypertensive medication 14. Fasting venous blood samples were obtained and analyzed following institutional laboratory protocols 6. All data extraction procedures were conducted independently by two blinded investigators, and discrepancies were resolved through adjudication by a third investigator. Data were fully anonymized in accordance with institutional and national data protection regulations. The study protocol complied with the principles outlined in the Declaration of Helsinki and received approval from the hospital's institutional ethics committee.

### 2.4 Preprocedural management

Prior to catheter ablation, all patients underwent transesophageal echocardiography (TEE) and cardiac computed tomography angiography (CTA) to exclude left atrial thrombus and evaluate pulmonary vein anatomy. Antiarrhythmic drugs were discontinued at least one week before the procedure. Stroke and bleeding risks were assessed using the CHA_2_DS_2_-VASc and HAS-BLED scores, respectively. Oral anticoagulation was managed according to standard clinical protocols 15, 16.

### 2.5 Ablation procedure

All procedures were performed under intravenous sedation. RFA was guided by either the CARTO or Ensite three-dimensional electroanatomical mapping systems. CBA was performed using a dedicated cryoballoon catheter following selective pulmonary vein angiography. Systemic anticoagulation was achieved with intravenous heparin to maintain an activated clotting time (ACT) between 300 and 400 seconds throughout the procedure. The detailed ablation techniques for RFA and CBA have been previously described 6, 15, 16. All procedures were performed by experienced electrophysiologists.

### 2.6 Postprocedural management and follow-up

Postprocedural echocardiography was performed to exclude pericardial tamponade. Oral anticoagulation therapy was resumed immediately after the procedure and continued for at least two months. Antiarrhythmic drugs were prescribed during the three-month blanking period to prevent early recurrence of AF. Patients were scheduled for follow-up visits at 3, 6, and 12 months post-ablation, and every six months thereafter. AF recurrence was defined as any documented episode lasting > 30 seconds, confirmed by either standard 12-lead electrocardiogram or wearable monitoring devices, occurring more than three months after the procedure.

### 2.7 Statistical analysis

Baseline characteristics were compared across NLR quartiles (Q1-Q4). Continuous variables were expressed as mean ± standard deviation and compared using one-way analysis of variance (ANOVA), while categorical variables were presented as frequencies and percentages and analyzed using the chi-square test. Missing data > 10% were excluded from analysis; variables with ≤ 10% missing values were imputed using multiple imputation. Extreme values were winsorized at the 1st and 99th percentiles. Kaplan-Meier (K-M) survival analysis was used to estimate recurrence-free survival, and log-rank tests were applied for intergroup comparisons. Multivariate Cox proportional hazards regression was used to assess the prognostic value of NLR, with variables with *P* < 0.05 in univariate analyses and other clinically relevant covariates (Supplementary Table 1). Multicollinearity was evaluated using the variance inflation factor, with a threshold < 5 considered acceptable 17 (Supplementary Table 2). Three models were constructed:

- Model 1: unadjusted.
- Model 2: adjusted for age, sex, smoking status, mean SBP, mean DBP, body mass index (BMI), New York Heart Association (NYHA) class, CHA_2_DS_2_-VASc score, HAS-BLED score, and ablation type.
- Model 3: further adjusted for coronary heart disease (CHD), diabetes mellitus (DM), white blood cell count (WBC), albumin (ALB), estimated glomerular filtration rate (eGFR), CRP, high-sensitivity C-reactive protein (hsCRP), N-terminal pro-B-type natriuretic peptide (NT-pro BNP), triglyceride (TG), left atrial diameter (LAD), and left ventricular ejection fraction (LVEF).

Restricted cubic spline (RCS) analysis was used to evaluate the non-linear relationship between NLR and AF recurrence. The RCS curve identified an inflection point at an NLR of 2.37, above which the risk of AF recurrence increased. This spline-derived threshold was then incorporated into the two-piecewise Cox model to quantify differential effects on either side of the inflection point and was also applied in downstream subgroup and joint analyses to maintain consistency with the biologically derived cutoff. Receiver operating characteristic (ROC) curves were used to compared the predictive accuracy of NLR, CRP, and hsCRP for AF recurrence risk, with areas under the curve (AUCs) compared using the DeLong test. ROC analysis further identified an optimal predictive cutoff of 2.61 based on the Youden index. This threshold was applied in classification-oriented evaluations, including the K-M survival curves and analyses of discriminative performance.

To evaluate the joint and interactive effects of NLR (based on the inflection point: < 2.37 vs. ≥ 2.37) and BP control status (SBP/DBP: < 140/90 mmHg vs. ≥ 140/90 mmHg) on AF recurrence, three additive interaction metrics were calculated: relative excess risk due to interaction (RERI), attributable proportion (AP), and synergy index (SI). The formulas for these metrics are as follows: *R*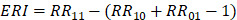; 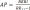; and 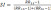. Where *RR*_11_ denotes the relative risk for individuals exposed to both the NLR ≥ 2.37 and BP control status (SBP/DBP ≥ 140/90 mmHg), *RR*_10_ represents the relative risk for individuals exposed solely to BP control status, and *RR*_01_ refers to the relative risk for individuals exposed solely to the NLR. The 95% confidence intervals (CIs) for these estimates were calculated using the delta method, as described by Hosmer and Lemeshow 18. The absence of additive interaction is indicated when the 95% CIs for RERI, AP, and SI include 0, 0, or 1, respectively. To assess multiplicative interaction, the following formula was applied: 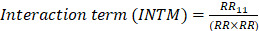. A 95% CI for INTM > 1 indicates a statistically significant interaction. Additionally, a Cox proportional hazards model was used to evaluate the joint effect of the NLR and BP control status on AF recurrence after ablation.

Several sensitivity analyses were conducted to test the robustness of the findings. First, to assess the potential impact of age on AF recurrence, we performed analyses among patients older than 40 years to determine whether this subgroup modification affected the overall results. Second, to account for the potential influence of extreme BMI on the risk of AF recurrence, we excluded patients with low or high BMI and reassessed the effects on outcomes. Third, potential confounding factors arising from lifestyle and comorbid conditions were considered by excluding smokers, alcohol consumers, individuals with CHD, DM, poor cardiac function (NYHA class III), and those with renal insufficiency (eGFR < 60 ml/min/1.73 m^2^). Finally, sensitivity analyses were performed among patients with high stroke risk and low bleeding risk on the basis of the CHA_2_DS_2_-VASc and HAS-BLED scores, to assess the impact of stroke risk on the relationship between the NLR and AF recurrence.

Mediation analysis was performed to determine whether the relationship between NLR and AF recurrence was mediated by LAD, LVEF, or markers of systemic inflammation. The NLR was treated as the independent variable, AF recurrence as the outcome, and each candidate mediator was evaluated within the mediation model framework. All the statistical analyses were conducted using R (version 4.3.2, R Foundation for Statistical Computing), Free Statisticals analysis platform (version 2.0; http://www.clinicalscientists.cn/freestatistics), and Zstats (version 1.0; www.zstats.net). A two-tailed *P* value < 0.05 was considered statistically significant.

## 3. Results

### 3.1 Baseline characteristics

The mean follow-up duration was 11.12 ± 2.07 months. A total of 46 patients (9.91%) were lost to follow-up, including 13 deaths from non-cardiovascular causes. Baseline characteristics of the 418 hypertensive patients with paroxysmal non-valvular AF are summarized in Table 1. The cohort had a mean age of 62.53 ± 9.04 years, with 39.23% being male. The mean NLR values in quartiles Q1 through Q4 were 1.34, 2.06, 2.74, and 4.60, respectively. A history of alcohol consumption and smoking was reported in 16.99% and 27.03% of participants, respectively. The mean CHA_2_DS_2_-VASc and HAS-BLED scores were 2.49 ± 1.69 and 1.42 ± 1.05, respectively. Notably, while the mean CHA_2_DS_2_-VASc score in Q2 was numerically lower compared with Q1, it subsequently increased in Q3 and Q4. A similar trend was observed for the HAS-BLED score. These patterns suggest a possible non-linear relationship between NLR levels and thromboembolic or bleeding risk burden. The prevalence of DM and CHD was 63.16% and 28.71%, respectively. BP was generally well controlled, with a mean SBP of 134.23 ± 18.56 mmHg and DBP of 81.64 ± 12.78 mmHg. Regarding antihypertensive medication use, calcium channel blockers (CCBs) were prescribed to 74.40% of patients, followed by angiotensin-converting enzyme inhibitors (ACEIs) or angiotensin II receptor blockers (ARBs) (44.26%). Additionally, 28.47% received angiotensin receptor-neprilysin inhibitors (ARNIs), 24.16% received β-blockers, and 16.99% received diuretics. Significant intergroup differences across NLR quartiles were observed for WBC, NEUT, LYMPH, CRP, and high-density lipoprotein cholesterol (HDL-C) (*P* < 0.05 for all). However, no significant differences were found in baseline LAD, LVEF (*P* = 0.445; *P* = 0.575, respectively), or in the type of catheter ablation performed (*P* = 0.536).

### 3.2 Clinical outcomes associated with NLR and AF recurrence

**Figure 1:**
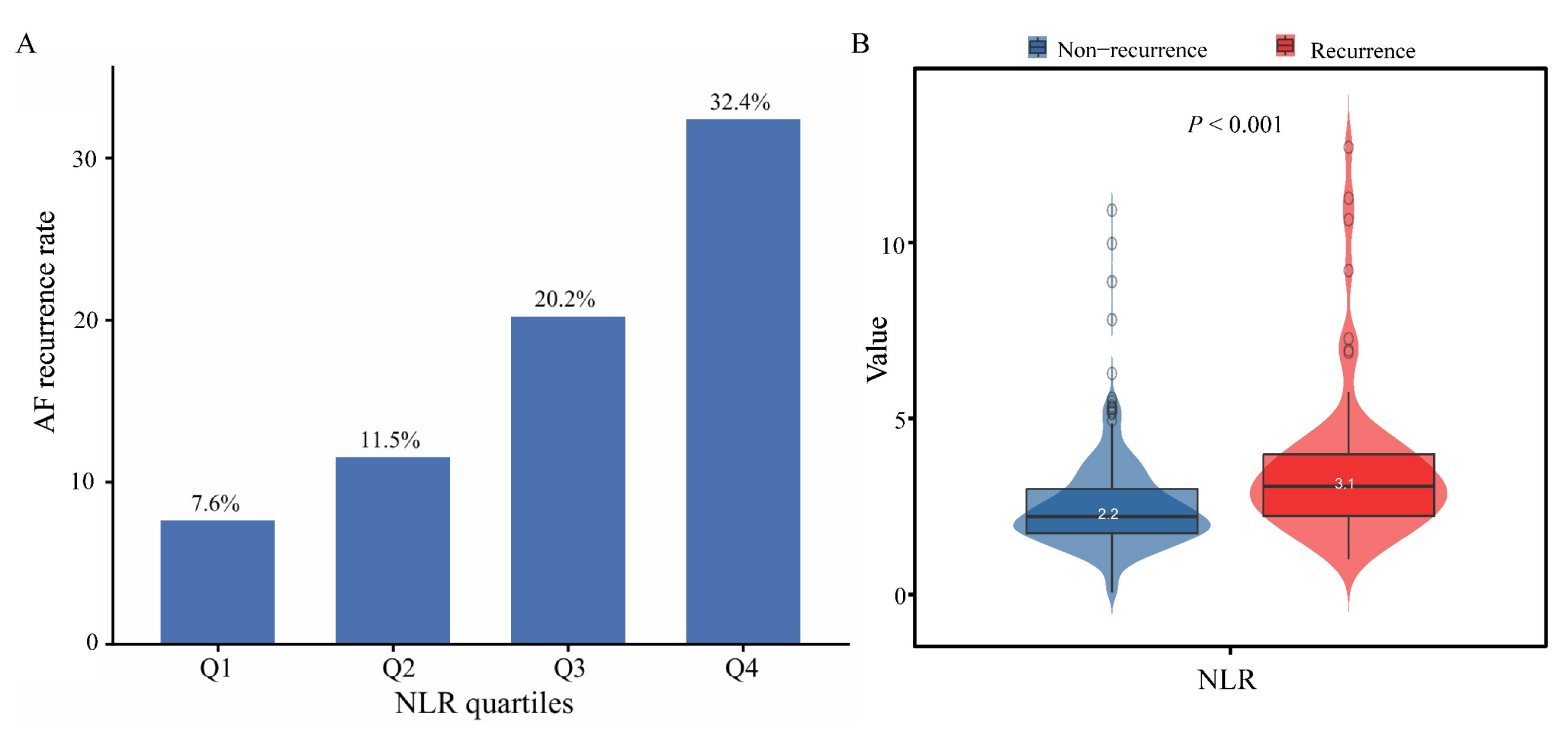
** AF recurrence rate across NLR quartiles (A) and distribution of NLR in the recurrence and non-recurrence groups (B).** *P* < 0.05 indicates statistical significance. Abbreviations: AF, atrial fibrillation; NLR, neutrophil-to-lymphocyte ratio.

**Figure 2:**
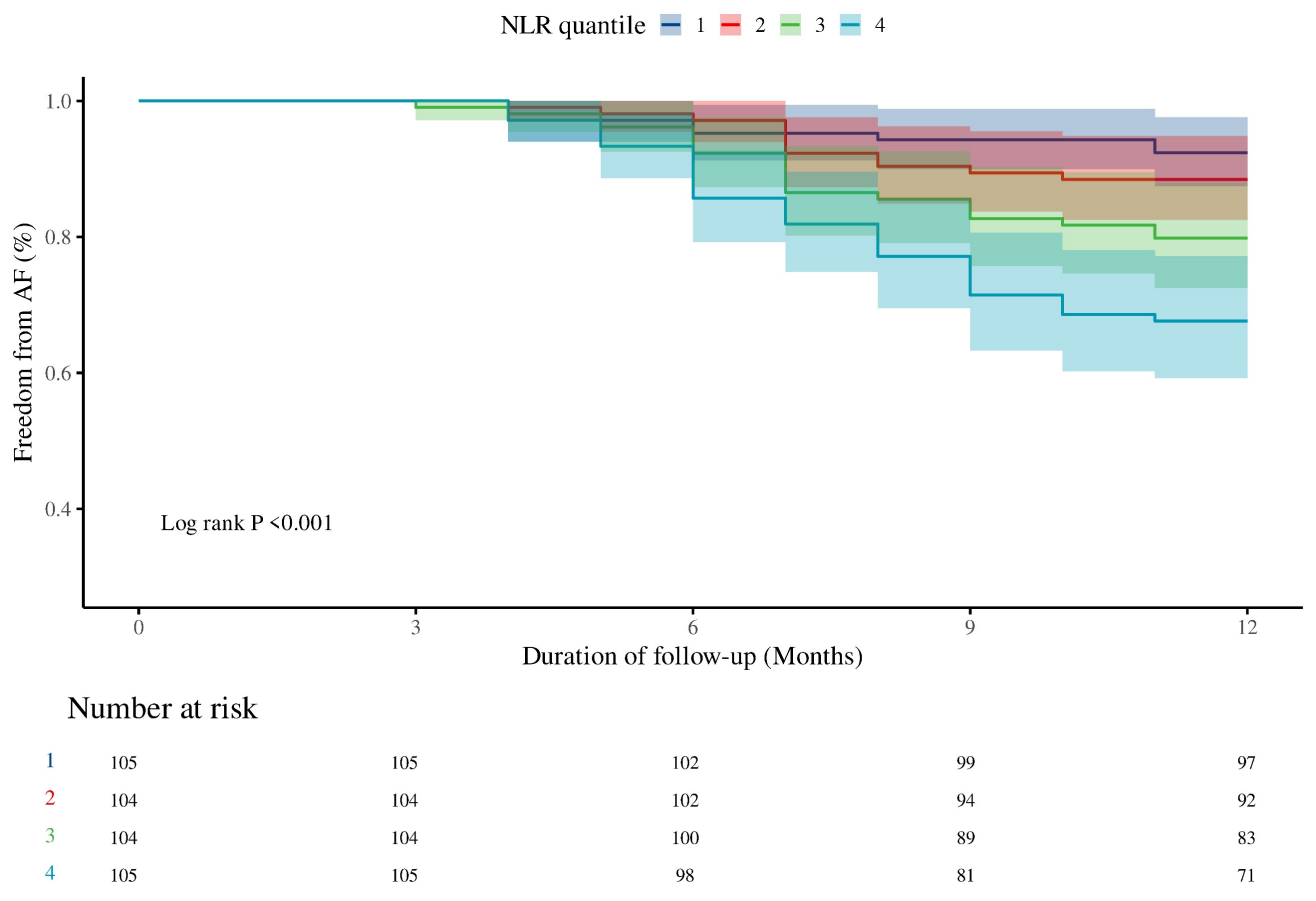
** K-M curves for AF recurrence stratified by NLR quartiles.** *P* < 0.05 indicates statistical significance. Abbreviations: AF, atrial fibrillation; K-M, Kaplan-Meier; NLR, neutrophil-to-lymphocyte ratio.

During the follow-up period, 75 patients (17.94%) experienced AF recurrence. As shown in Figure 1A, the proportion of AF recurrence increased across rising NLR quartiles (Q1-Q4). The mean NLR was significantly higher in the recurrence group than in the non-recurrence group (3.1 vs. 2.2, *P* < 0.001) (Figure 1B). K-M survival analysis revealed a significant association between NLR quartiles and AF recurrence, with the highest recurrence rate observed in Q4 (log-rank *P* < 0.001) (Figure 2). Contour plots further demonstrated that elevated SBP combined with high NLR was associated with increased recurrence risk. Notably, the presence of both high NLR and low DBP appeared to synergistically worsen outcomes (Supplementary Figure 2). Cox regression analysis results are summarized in Table 2. When modeled as a continuous variable, each 1-unit increase in NLR was associated with a 28% increased risk of AF recurrence [hazard ratio (HR): 1.28, 95% CI: 1.17-1.39; *P* < 0.001], and the association remained significant after full adjustment (HR: 1.30, 95% CI: 1.12-1.50; *P* < 0.001). In categorical analysis, NLR quartiles Q3 (HR: 2.82, 95% CI: 1.25-6.37) and Q4 (HR: 4.86, 95% CI: 2.25-10.51) were significantly associated with AF recurrence risk in Model 1. In Model 3, HRs for Q3 and Q4 were 2.40 (95% CI: 1.04-5.56) and 4.34 (95% CI: 1.90-9.95), respectively.

### 3.3 Non-linear relationship between NLR and AF recurrence

**Figure 3:**
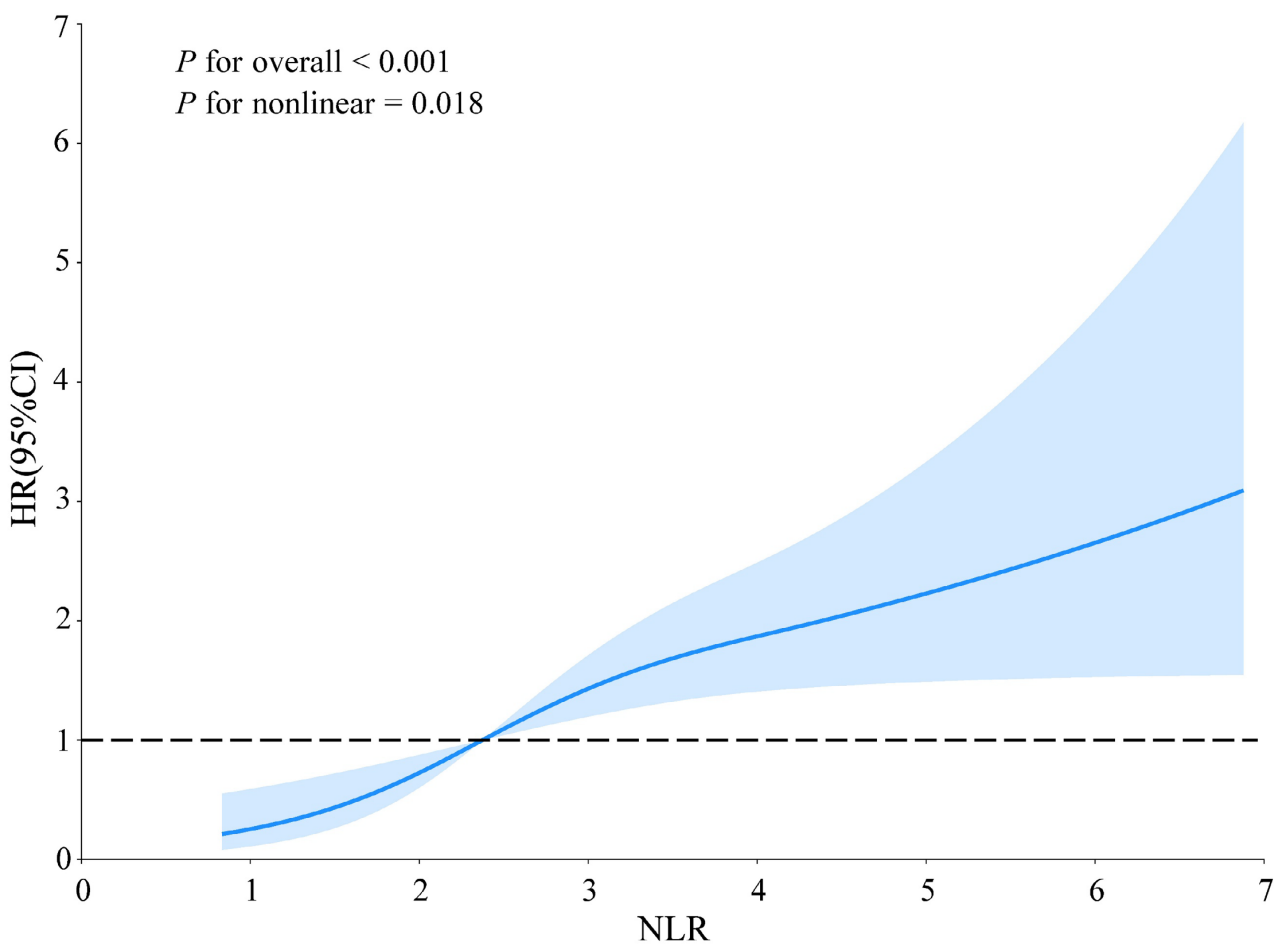
** Non-linear relationship between NLR and AF recurrence in hypertensive patients.** The solid line and blue area represent the estimated values and their corresponding 95% CIs, respectively. *P* < 0.05 indicates statistical significance. Abbreviations: AF, atrial fibrillation; CI, confidence interval; HR, hazard ratio; NLR, neutrophil-to-lymphocyte ratio.

RCS analysis identified a non-linear relationship between NLR and AF recurrence (non-linear *P* = 0.018) (Figure 3). A two-piecewise Cox regression model determined an inflection point at NLR = 2.37. Above this threshold, each unit increase in NLR was associated with a significantly higher risk of AF recurrence (HR: 1.20, 95% CI: 1.01-1.44; *P* = 0.042) (Table 3). Subgroup analyses demonstrated consistent positive associations between NLR and AF recurrence across all subgroups, with no significant interactions observed (Supplementary Figure 3). Specifically, the predictive value of NLR was similar in both sexes (*P* for interaction = 0.896) and across age groups (< 60 years vs. ≥ 60 years) (*P* for interaction = 0.423).

### 3.4 Predictive performance of NLR versus CRP and hsCRP

**Figure 4:**
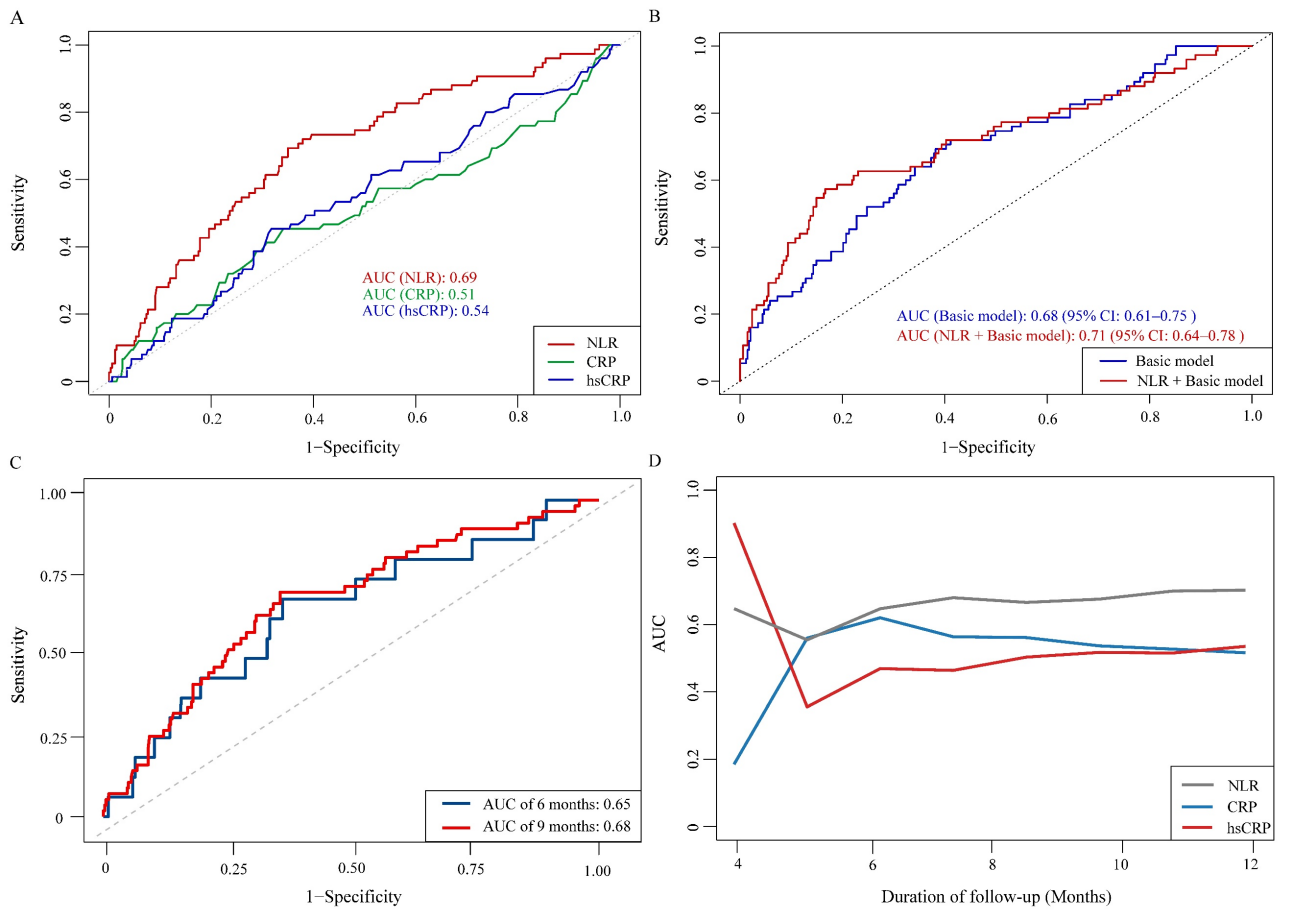
** ROC curves for evaluating the predictive value of NLR, CRP, and hsCRP for AF recurrence.** A: Comparison of NLR, CRP, and hsCRP in predicting AF recurrence. B: Incremental predictive value of NLR when added to the basic model. C: Time-dependent ROC curves for NLR in predicting AF recurrence. D: Time-dependent AUC curves for NLR, CRP, and hsCRP. Abbreviations: AF, atrial fibrillation; AUC, area under the curve; CRP, C-reactive protein; hsCRP, high-sensitivity C-reactive protein; NLR, neutrophil-to-lymphocyte ratio; ROC, receiver operating characteristic.

**Figure 5:**
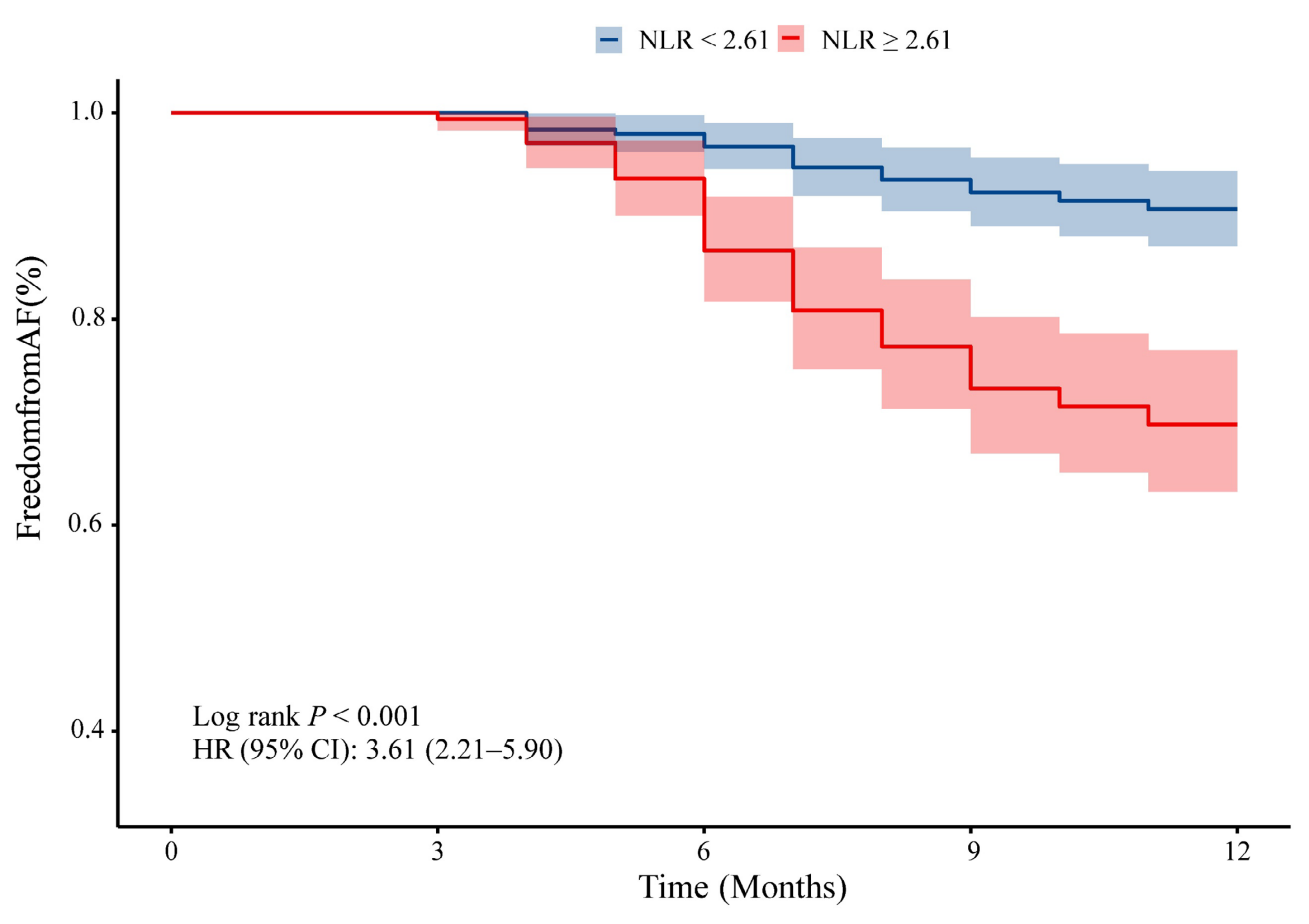
** K-M survival analysis for AF recurrence in hypertensive patients based on cutoff values.** Abbreviations: AF, atrial fibrillation; CI, confidence interval; HR, hazard ratio; K-M, Kaplan-Meier; NLR, neutrophil-to-lymphocyte ratio.

ROC curve analysis was performed to assess the prognostic value of NLR, CRP, and hsCRP for AF recurrence. The AUC for NLR was significantly higher than for CRP (0.69 vs. 0.51, *P* = 0.001) and hsCRP (0.69 vs. 0.54, *P* = 0.003) (Figure 4A, Supplementary Table 3). The optimal NLR cutoff value for predicting AF recurrence was 2.61 (Supplementary Table 3). Patients were thus categorized into high NLR (≥ 2.61) and low NLR (< 2.61) groups. K-M survival analysis revealed that patients with high NLR had a significantly increased risk of AF recurrence (HR: 3.61, 95% CI: 2.21-5.90, log-rank *P* < 0.001) (Figure 5). Although the addition of NLR to the baseline model increased the AUC from 0.68 to 0.71, the improvement was not statistically significant (*P* = 0.100) (Figure 4B). Time-dependent ROC analyses indicated that NLR maintained moderate predictive value for AF recurrence at both 6 months (AUC: 0.65) and 9 months (AUC: 0.68) (Figure 4C). In contrast, hsCRP had better short-term predictive value but declined over time, while NLR's predictive value remained stable, surpassing that of both CRP and hsCRP over the follow-up period (Figure 4D).

### 3.5 Interaction and joint effects of NLR and BP control

Interaction analysis revealed that the 95% CIs for RERI, AP, and SI were -0.47 (-3.34-2.41), -0.12 (-0.86-0.63), and 0.87 (0.36-2.08), respectively, indicating that there was no significant additive interaction between the NLR and BP control status. For multiplicative interaction, the INTM was 0.57 (95%CI: 0.19-1.66), indicating no significant interaction between NLR and BP control on AF recurrence (Table 4). Joint analysis demonstrated differential effects depending on the combination of NLR level and BP control status. Compared with the groups with an NLR < 2.37 and controlled hypertension, the groups with an NLR < 2.37 and uncontrolled hypertension did not show a significant increase in AF recurrence risk (HR: 1.82, 95% CI: 0.73-4.53; *P* = 0.200). However, for patients with an NLR ≥ 2.37 and controlled hypertension, the risk of AF recurrence was significantly elevated (HR: 3.55, 95% CI: 1.67-7.53; *P* < 0.001). Moreover, in the group with an NLR ≥ 2.37 and uncontrolled hypertension, the risk of AF recurrence was also significantly increased (HR: 3.92, 95% CI: 1.82-8.42; *P* < 0.001) (Table 4).

### 3.6 Sensitivity analyses

Sensitivity analyses evaluating the joint association of NLR and BP control with AF recurrence revealed no substantial changes across subgroups. Compared with the NLR < 2.37 and controlled hypertension groups, the risk of AF recurrence was slightly greater in the subgroup with an NLR ≥ 2.37 and controlled hypertension after individuals with underweight or obesity (HR: 3.75, 95% CI: 1.62-8.69), alcohol consumption (HR: 3.58, 95% CI: 1.46-8.83), and NYHA functional class I or II (HR: 3.63, 95% CI: 1.57-8.39) were excluded. Conversely, the risk of AF recurrence was slightly reduced but remained significant in the other subgroup. Additionally, compared with the NLR < 2.37 and controlled hypertension groups, a slightly greater risk of AF recurrence was observed in the subgroup with NLR ≥ 2.37 and uncontrolled hypertension among middle-aged patients (HR: 4.02, 95% CI: 1.57-10.32), those with LAD < 50 mm (HR: 3.98, 95% CI: 1.55-10.21), those with NYHA functional class I or II (HR: 4.07, 95% CI: 1.75-9.47), and those with low bleeding risk (HR: 4.95, 95% CI: 1.81-13.57). Across all other subgroups, the risk of AF recurrence was slightly lower but remained statistically significant. The detailed results of the sensitivity analyses are provided in Table 5.

### 3.7 Mediation analysis

Mediation analysis revealed that the average direct effects (ADEs) of several demographic, metabolic, and inflammatory markers on the association between NLR and AF recurrence were statistically significant (*P* < 0.001), indicating that these variables independently influenced recurrence risk. However, their mediation effects were minimal. The average causal mediation effects (ACMEs) for these mediators were not statistically significant, indicating that they did not exert a significant indirect effect on the relationship between the NLR and AF recurrence. Detailed mediation results are presented in Supplementary Table 4.

## 4. Discussion

This observational cohort study provides novel insights into the prognostic value of the NLR in predicting AF recurrence following catheter ablation in patients with hypertension. To the best of our knowledge, this is the first investigation to evaluate this association in a hypertensive population. The major findings are as follows: (i) the NLR was independently associated with post-ablation AF recurrence; (ii) a significant non-linear relationship between the NLR and AF recurrence was identified, with an inflection point at 2.37, beyond which AF recurrence risk increased markedly; (iii) NLR demonstrated superior prognostic performance for AF recurrence compared to CRP and hsCRP, maintaining consistent predictive performance over time, although it did not provide incremental predictive value when added to the basic model; (iv) no significant additive or multiplicative interactions were detected between NLR and BP control status on AF recurrence risk; (v) an NLR ≥ 2.37 was associated with a significant increase in the risk of AF recurrence in hypertensive patients, particularly those with uncontrolled hypertension. The combined presence of a high NLR and uncontrolled hypertension should be considered a high-risk marker for AF recurrence; (vi) sensitivity analyses confirmed that an NLR ≥ 2.37 consistently increased the risk of AF recurrence, particularly in patients with uncontrolled hypertension, across various subgroups; and (vii) demographic, metabolic, and inflammatory markers did not significantly mediate the relationship between NLR and AF recurrence.

The NLR is a simple, cost-effective marker of systemic inflammation and has garnered increasing interest as a cardiovascular disease (CVD) risk predictor. A retrospective cohort study demonstrated that elevated NLR is an independent predictor of both all-cause mortality (HR 1.46, 95% CI 1.30-1.62; *P* < 0.0001) and cardiovascular mortality (HR 1.58, 95% CI 1.33-1.82; *P* < 0.0001) in patients with CHD and hypertension 19. Similarly, a cross-sectional study by Wang et al. 20 indicated a significant independent association between the NLR and high-risk coronary artery disease [odds ratio (OR) = 1.74, 95% CI: 1.30-2.33; *P* = 0.0002]. Furthermore, a meta-analysis revealed that NLR > 2.1 serves as an independent predictor of in-hospital mortality (HR 1.13, 95% CI 1.01-1.27) and long-term all-cause mortality (HR 1.43, 95% CI 1.10-1.85) in heart failure patients 21. These findings underscore the potential of the NLR as an effective marker for predicting CVD risk. On the basis of these previous studies, our research demonstrated that the NLR is an independent risk factor for AF recurrence following catheter ablation in hypertensive patients, with a significant non-linear relationship between the NLR and AF recurrence. Notably, an inflection point at an NLR of 2.37 was identified, above which the risk of recurrence significantly increased. Given that patients with an NLR above 2.37 have a greater risk of AF recurrence, closer monitoring and potentially more aggressive therapeutic interventions are warranted.

Hypertension is a major risk factor for AF recurrence after ablation, and effective BP control is crucial in managing hypertensive patients with AF 22. However, our findings suggest that BP control alone may not significantly reduce the risk of AF recurrence in patients with a high NLR. Specifically, compared with the NLR < 2.37 in the controlled hypertension group, the NLR < 2.37 in the uncontrolled hypertension group did not significantly increase the risk of AF recurrence (*P* = 0.200). In contrast, regardless of BP control, the risk of AF recurrence was significantly greater in the NLR ≥ 2.37 group (*P* < 0.001). Therefore, an NLR ≥ 2.37 serves as an independent risk factor for AF recurrence, irrespective of BP control status. Notably, patients with both a high NLR and uncontrolled hypertension had the highest risk of AF recurrence (HR: 3.92, 95% CI: 1.82-8.42). This dual burden of heightened inflammation and suboptimal BP control may synergistically increase AF susceptibility, suggesting that these patients may benefit from more intensive treatment strategies. In patients with elevated NLRs, an exacerbated inflammatory environment promotes atrial remodeling, increasing the susceptibility of the atrium to arrhythmogenic triggers 23. Simultaneously, uncontrolled hypertension induces atrial structural changes, including fibrosis and dilation, further increasing the likelihood of atrial remodeling 24. These mechanical and electrical alterations create a substrate conducive to AF. While BP control remains crucial in clinical management, addressing the inflammatory component is equally important for effectively reducing the risk of AF recurrence. Anti-inflammatory strategies, in conjunction with standard antihypertensive therapies, may offer a more comprehensive approach to managing AF recurrence. For example, combining colchicine, antioxidants, or dietary interventions aimed at reducing systemic inflammation with antihypertensive therapies could help address the underlying inflammatory processes 25-27. Larger-scale prospective studies are needed to further validate the effectiveness of these combined approaches.

ROC analysis demonstrated that the NLR outperforms the CRP and hsCRP levels in the prediction of AF recurrence, particularly in the long term. This finding is consistent with previous studies, which suggest that the NLR may be a more reliable and stable biomarker for CVDs 28-30. While CRP and hsCRP are valuable for detecting acute inflammation, their predictive value for long-term outcomes, such as AF recurrence, may be limited. In contrast, the ability of the NLR to maintain predictive accuracy over time suggests that it could serve as a superior long-term marker of systemic inflammation, which is critical for managing AF recurrence. Moreover, when the NLR was added to the basic model, it did not provide incremental predictive value, indicating that while the NLR is an important predictor, its contribution to model discrimination may be limited. Nevertheless, when interpreted alongside other clinical parameters such as BP control status, the NLR offers complementary biological information that may help contextualize AF recurrence risk.

At the molecular level, the association between the NLR and AF recurrence may involve multiple inflammatory pathways. Elevated NEUT in patients with high NLRs results in the release of various proinflammatory cytokines, chemokines, and matrix metalloproteinases, which promote atrial remodeling and fibrosis 31. These inflammatory mediators disrupt normal atrial conduction, creating an environment conducive to AF. NEUT also exacerbates endothelial dysfunction, promotes thrombogenesis, and amplifies the inflammatory cascade 32. Furthermore, NEUT has been shown to increase the production of reactive oxygen species, which can cause cellular damage and further promote atrial structural remodeling, thereby fostering arrhythmogenesis 33. The reduction in LYMPH in patients with high NLRs may also contribute to this process. LYMPH plays a crucial role in modulating inflammatory responses and facilitating tissue repair. When LYMPH are diminished, the resolution of inflammation is impaired, leading to a prolonged inflammatory cycle that fosters continued atrial remodeling 34. This sustained neutrophilic inflammation and impaired LYMPH-mediated resolution may represent key mechanisms underlying AF recurrence in these patients. Moreover, hypertension further complicates this inflammatory process by inducing structural changes in the atria, including fibrosis and atrial dilation, which create a substrate for AF. Elevated BP places additional mechanical strain on the atrial myocardium, and when combined with the inflammatory changes induced by a high NLR, it exacerbates the remodeling process and promotes AF recurrence 35. The pathophysiological relationship between inflammation and AF recurrence in the context of an elevated NLR may be further complicated by comorbidities such as DM, obesity, or metabolic syndrome, which are common in hypertensive patients and are associated with increased systemic inflammation. Future studies should explore the interplay between the NLR, metabolic risk factors, and atrial remodeling to better understand the full spectrum of mechanisms driving AF recurrence in this patient population.

## 5. Limitations

Several limitations should be acknowledged. First, its retrospective design limits the ability to establish causal relationships, particularly regarding the effect of NLR on AF recurrence. To confirm these findings, large-scale prospective studies with long-term follow-up are necessary. Second, the study was conducted at a single center, which may reduce the generalizability of the results. Third, we did not examine the impact of specific anti-inflammatory treatments on patient outcomes. Future clinical trials focusing on targeted anti-inflammatory therapies may provide valuable insights into their potential role in AF recurrence. Fourth, the lack of standardized protocols for detecting arrhythmia recurrence (e.g., implantable loop recorders) may have led to an underestimation of AF recurrence rates. Fifth, this study primarily included patients with paroxysmal non-valvular AF and hypertension, excluding those with persistent AF. Therefore, the results may not be applicable to the broader hypertensive AF population, and further research is needed to evaluate the predictive value of NLR in persistent AF patients. Sixth, hormonal fluctuations in female patients may affect NEUT and LYMPH levels, but we did not account for this variable. Future studies should control for hormonal fluctuations to better assess their impact on the predictive value of NLR for AF recurrence. Finally, the number of covariates included in the multivariate models was relatively high compared with the number of AF recurrence events, which may affect the precision and stability of the estimates.

## 6. Conclusion

In conclusion, this study demonstrated that the NLR is an independent predictor of recurrence after catheter ablation in hypertensive patients with paroxysmal AF. A non-linear relationship was identified between the NLR and AF recurrence, with an inflection point at an NLR ≥ 2.37, beyond which the risk of recurrence increases significantly. Compared with traditional inflammatory markers, the NLR provides a more consistent and stable prognostic value. Moreover, regardless of BP control, patients with an NLR ≥ 2.37 are associated with an increased risk of AF recurrence, particularly those with uncontrolled hypertension. The combination of a high NLR and uncontrolled hypertension represents a high-risk marker for AF recurrence. These findings underscore the potential of the NLR as an efficient biomarker for AF recurrence risk stratification in hypertensive patients and suggest that anti-inflammatory strategies, when combined with BP control, may effectively reduce the risk of AF recurrence. Further prospective studies are needed to confirm these findings and evaluate the efficacy of targeted anti-inflammatory therapies.

## Supplementary Material

Supplementary figures and tables.

## Figures and Tables

**Table 1:** Baseline characteristics of the four groups based on NLR quartiles.

Variables	Total	Q1 (< 1.80)	Q2 [1.80-2.37)	Q3 [2.37-3.22)	Q4 (≥ 3.22)	*P* value
	N = 418	105	104	104	105	
NLR	2.69 ± 1.55	1.34 ± 0.40	2.06 ± 0.16	2.74 ± 0.24	4.60 ± 1.84	**< 0.001**
Demographics						
Age (y)	62.53 ± 9.04	62.11 ± 9.19	61.01 ± 8.57	63.34 ± 8.60	63.65 ± 9.62	0.132
Male (n, %)	164 (39.23)	30 (28.57)	41 (39.42)	48 (46.15)	45 (42.86)	0.053
Basic information						
Alcohol consumption (n, %)	71 (16.99)	15 (14.29)	20 (19.23)	20 (19.23)	16 (15.24)	0.679
Smoking status (n, %)	113 (27.03)	17 (16.19)	33 (31.73)	32 (30.77)	31 (29.52)	**0.037**
Mean SBP (mmHg)	134.23 ± 18.56	135.02 ± 19.59	133.23 ± 17.70	134.88 ± 18.63	133.79 ± 18.46	0.879
Mean DBP (mmHg)	81.64 ± 12.78	81.81 ± 12.74	82.84 ± 12.30	79.73 ± 11.79	82.18 ± 14.12	0.330
BMI (kg/m^2^)	24.35 ± 3.03	24.10 ± 2.78	24.31 ± 3.22	24.53 ± 2.96	24.46 ± 3.17	0.743
NYHA classification						0.883
I	110 (26.32)	27 (25.71)	27 (25.96)	26 (25.00)	30 (28.57)	
II	266 (63.64)	71 (67.62)	65 (62.50)	66 (63.46)	64 (60.95)	
III	42 (10.05)	7 (6.67)	12 (11.54)	12 (11.54)	11 (10.48)	
CHA_2_DS_2_-VASc score	2.49 ± 1.69	2.30 ± 1.53	2.17 ± 1.59	2.76 ± 1.88	2.74 ± 1.68	**0.018**
HAS-BLED score	1.42 ± 1.05	1.37 ± 0.97	1.17 ± 1.00	1.58 ± 1.13	1.57 ± 1.04	**0.014**
Comorbidities						
DM (n, %)	264 (63.16)	70 (66.67)	55 (52.88)	68 (65.38)	71 (67.62)	0.094
CHD (n, %)	120 (28.71)	27 (25.71)	28 (26.92)	33 (31.73)	32 (30.48)	0.742
Pharmacotherapy						
ACEI/ARB (n, %)	185 (44.26)	39 (37.14)	51 (49.04)	41 (39.42)	54 (51.43)	0.098
ARNI (n, %)	119 (28.47)	37 (35.24)	27 (25.96)	28 (26.92)	27 (25.71)	0.362
β-blockers (n, %)	101 (24.16)	37 (35.24)	24 (23.08)	21 (20.19)	19 (18.10)	**0.018**
CCBs (n, %)	311 (74.40)	79 (75.24)	81 (77.88)	77 (74.04)	74 (70.48)	0.669
Diuretics (n, %)	71 (16.99)	16 (15.24)	18 (17.31)	18 (17.31)	19 (18.10)	0.953
Laboratory data						
WBC (10^9^/L)	6.20 ± 1.76	5.45 ± 1.33	5.86 ± 1.29	6.12 ± 1.67	7.37 ± 2.05	**< 0.001**
Hb (g/L)	134.00 ± 16.09	131.99 ± 16.70	135.32 ± 16.68	134.50 ± 15.32	134.22 ± 15.63	0.485
RBC (10^12^/L)	4.46 ± 0.60	4.36 ± 0.57	4.50 ± 0.61	4.47 ± 0.59	4.51 ± 0.61	0.210
PLT (10^9^/L)	197.04 ± 55.95	194.42 ± 54.72	193.86 ± 53.06	207.03 ± 63.04	192.93 ± 51.94	0.217
NEUT (10^9^/L)	3.99 ± 1.58	2.78 ± 1.03	3.57 ± 0.82	4.08 ± 1.16	5.51 ± 1.71	**< 0.001**
LYMPH (10^9^/L)	1.71 ± 1.23	2.34 ± 2.23	1.74 ± 0.39	1.49 ± 0.40	1.26 ± 0.34	**< 0.001**
ALB (g/dL)	38.77 ± 3.37	39.00 ± 3.32	38.57 ± 2.56	38.55 ± 3.83	38.97 ± 3.66	0.648
eGFR (ml/min/1.73m^2^)	85.03 ± 26.56	87.98 ± 27.36	86.34 ± 23.99	86.83 ± 28.64	79.00 ± 25.44	0.058
CRP (mg/L)	5.10 ± 9.68	4.42 ± 8.70	2.92 ± 2.95	5.47 ± 10.03	7.57 ± 13.44	**0.005**
hsCRP (mg/L)	1.77 ± 3.33	1.85 ± 4.13	1.63 ± 3.47	1.88 ± 2.77	1.70 ± 2.80	0.942
NT-pro BNP (pg/mL)	406.18 ± 502.31	354.54 ± 365.99	469.98 ± 681.98	373.01 ± 435.48	427.50 ± 470.08	0.331
FPG (mmol/L)	4.92 ± 1.09	4.94 ± 1.19	4.88 ± 0.84	4.91 ± 1.20	4.96 ± 1.09	0.954
HbA1c (%)	6.22 ± 0.85	6.04 ± 0.66	6.26 ± 0.84	6.28 ± 0.97	6.31 ± 0.89	0.074
TG (mmol/L)	1.61 ± 1.12	1.40 ± 0.65	1.65 ± 1.22	1.79 ± 1.26	1.61 ± 1.23	0.092
TC (mmol/L)	3.99 ± 0.93	4.11 ± 0.98	4.03 ± 0.95	3.87 ± 0.95	3.95 ± 0.85	0.268
HDL-C (mmol/L)	1.14 ± 0.29	1.19 ± 0.30	1.12 ± 0.26	1.08 ± 0.29	1.16 ± 0.29	**0.029**
LDL-C (mmol/L)	2.40 ± 0.79	2.54 ± 0.82	2.42 ± 0.74	2.32 ± 0.84	2.35 ± 0.73	0.186
Echocardiographic data						
LAD (mm)	37.54 ± 5.01	37.12 ± 5.14	37.81 ± 4.90	37.16 ± 4.80	38.05 ± 5.21	0.445
LVDd (mm)	47.85 ± 4.11	47.55 ± 3.91	48.27 ± 3.71	47.63 ± 3.85	47.94 ± 4.88	0.580
RAD (mm)	33.43 ± 4.50	32.95 ± 4.14	33.88 ± 4.51	33.34 ± 4.57	33.56 ± 4.78	0.508
RVDd (mm)	31.33 ± 3.30	31.01 ± 3.25	31.74 ± 3.03	31.35 ± 3.67	31.21 ± 3.23	0.436
LVEF (%)	60.54 ± 5.49	60.91 ± 5.24	60.00 ± 5.25	60.88 ± 5.55	60.36 ± 5.90	0.575
Type of ablation						0.536
RFA (n, %)	146 (34.93)	32 (30.48)	40 (38.46)	34 (32.69)	40 (38.10)	
CBA (n, %)	272 (65.07)	73 (69.52)	64 (61.54)	70 (67.31)	65 (61.90)	

The values are presented as the means ± standard deviations or n (%). Bold values indicate statistical significance. A *P* value < 0.05 indicated a significant difference. Abbreviations: ACEI, angiotensin-converting enzyme inhibitor; ALB, albumin; ARB, angiotensin II receptor blocker; ARNI, angiotensin receptor-neprilysin inhibitor; BMI, body mass index; CBA, cryoballoon ablation; CCB, calcium channel blocker; CHD, coronary heart disease; CRP, C-reactive protein; DBP, diastolic blood pressure; DM, diabetes mellitus; eGFR, estimated glomerular filtration rate; FPG, fasting plasma glucose; Hb, hemoglobin; HbA1c, glycosylated hemoglobin; HDL-C, high-density lipoprotein cholesterol; hsCRP, high-sensitivity C-reactive protein; LAD, left atrial diameter; LDL-C, low-density lipoprotein cholesterol; LVDd, left ventricular end diastolic diameter; LVEF, left ventricular ejection fraction; LYMPH, lymphocytes count; NEUT, neutrophils count; NLR, neutrophil-to-lymphocyte ratio; NT-pro BNP, N-terminal pro-B-type natriuretic peptide; NYHA, New York Heart Association; PLT, platelet; RAD, right atrial diameter; RBC, red blood cell count; RFA, radiofrequency ablation; RVDd, right ventricular end diastolic diameter; SBP, systolic blood pressure; TC, total cholesterol; TG, triglyceride; WBC, white blood cell count.

**Table 2:** Cox regression models for the association between NLR and AF recurrence after ablation in hypertensive patients.

	Continuous variable	Quantiles of the NLR				*P* for trend
		Q1	Q2	Q3	Q4	
AF recurrence						
Model 1 HR (95% CI) *P* value	**1.28 (1.17-1.39) < 0.001**	1(Ref)	1.54 (0.63-3.76) 0.346	**2.82 (1.25-6.37) 0.012**	**4.86 (2.25-10.51) < 0.001**	**< 0.001**
Model 2 HR (95% CI) *P* value	**1.29 (1.18-1.42) < 0.001**	1(Ref)	1.44 (0.58-3.56) 0.426	**2.59 (1.14-5.89) 0.023**	**4.91 (2.25-10.72) < 0.001**	**< 0.001**
Model 3 HR (95% CI) *P* value	**1.30 (1.12-1.50) < 0.001**	1(Ref)	1.30 (0.52-3.28) 0.572	**2.40 (1.04-5.56) 0.041**	**4.34 (1.90-9.95) 0.001**	**< 0.001**

Bold values indicate statistical significance. A *P* value < 0.05 indicated a significant difference. Model 1: Unadjusted. Model 2: Adjusted for age, sex, smoking status, mean SBP, mean DBP, BMI, NYHA classification, CHA_2_DS_2_-VASc score, HAS-BLED score, and type of ablation. Model 3: Adjusted for age, sex, smoking status, mean SBP, mean DBP, BMI, NYHA classification, CHA_2_DS_2_-VASc score, HAS-BLED score, type of ablation, CHD, DM, WBC, ALB, eGFR, CRP, hsCRP, NT-pro BNP, TG, LAD, and LVEF. Abbreviations: AF, atrial fibrillation; ALB, albumin; BMI, body mass index; CHD, coronary heart disease; CI, confidence interval; CRP, C-reactive protein; DBP, diastolic blood pressure; DM, diabetes mellitus; eGFR, estimated glomerular filtration rate; HR, hazard ratio; hsCRP, high-sensitivity C-reactive protein; LAD, left atrial diameter; LVEF, left ventricular ejection fraction; NLR, neutrophil-to-lymphocyte ratio; NT-pro BNP, N-terminal pro-B-type natriuretic peptide; NYHA, New York Heart Association; SBP, systolic blood pressure; TG, triglyceride; WBC, white blood cell count.

**Table 3:** Threshold effect analysis of NLR on AF recurrence.

	Adjusted HR (95% CI)	*P* value
AF recurrence		
Fitting by two-piecewise Cox proportional risk model		
Inflection point	2.37	
NLR < 2.37	1.81 (0.59-5.59)	0.301
NLR ≥ 2.37	**1.20 (1.01-1.44)**	**0.042**
Likelihood ratio test		**< 0.001**

The model was adjusted for age, sex, smoking status, mean SBP, mean DBP, BMI, NYHA classification, CHA_2_DS_2_-VASc score, HAS-BLED score, type of ablation, CHD, DM, WBC, ALB, eGFR, CRP, hsCRP, NT-pro BNP, TG, LAD, and LVEF. Bold values indicate statistical significance. A *P* value < 0.05 indicated a significant difference. Abbreviations: AF, atrial fibrillation; ALB, albumin; BMI, body mass index; CHD, coronary heart disease; CI, confidence interval; CRP, C-reactive protein; DBP, diastolic blood pressure; DM, diabetes mellitus; eGFR, estimated glomerular filtration rate; HR, hazard ratio; hsCRP, high-sensitivity C-reactive protein; LAD, left atrial diameter; LVEF, left ventricular ejection fraction; NLR, neutrophil-to-lymphocyte ratio; NT-pro BNP, N-terminal pro-B-type natriuretic peptide; NYHA, New York Heart Association; SBP, systolic blood pressure; TG, triglyceride; WBC, white blood cell count.

**Table 4:** Interaction and joint analyses of the effects of NLR and BP control status on AF recurrence.

NLR group	BP control status	HR (95% CI) *P* value	RERI (95% CI)	AP (95% CI)	SI (95% CI)	INTM (95% CI)
< 2.37	Controlled	1 (Ref)				
< 2.37	Uncontrolled	1.82 (0.73-4.53) 0.200				
≥ 2.37	Controlled	**3.55 (1.67-7.53) < 0.001**				
≥ 2.37	Uncontrolled	**3.92 (1.82-8.42) < 0.001**				
			-0.47 (-3.34-2.41)	-0.12 (-0.86-0.63)	0.87 (0.36-2.08)	0.57 (0.19-1.66)

Adjusted for age, sex, smoking status, mean SBP, mean DBP, BMI, NYHA classification, CHA_2_DS_2_-VASc score, HAS-BLED score, type of ablation, CHD, DM, WBC, ALB, eGFR, CRP, hsCRP, NT-pro BNP, TG, LAD, and LVEF. Bold values indicate statistical significance. A *P* value < 0.05 indicated a significant difference. Abbreviations: AF, atrial fibrillation; ALB, albumin; AP, attributable proportion; BMI, body mass index; BP, blood pressure; CHD, coronary heart disease; CI, confidence interval; CRP, C-reactive protein; DBP, diastolic blood pressure; DM, diabetes mellitus; eGFR, estimated glomerular filtration rate; HR, hazard ratio; hsCRP, high-sensitivity C-reactive protein; INTM, interaction term; LAD, left atrial diameter; LVEF, left ventricular ejection fraction; NLR, neutrophil-to-lymphocyte ratio; NT-pro BNP, N-terminal pro-B-type natriuretic peptide; NYHA, New York Heart Association; RERI, relative excess risk due to interaction; SBP, systolic blood pressure; SI, synergy index; TG, triglyceride; WBC, white blood cell count.

**Table 5:** Sensitivity analyses of the joint effect of NLR and BP control status on AF recurrence.

Sensitivity condition	HR (95% CI) *P* value				
	NLR < 2.37 & Controlled hypertension	NLR < 2.37 & Uncontrolled hypertension	NLR ≥ 2.37 & Controlled hypertension	NLR ≥ 2.37 & Uncontrolled hypertension	*P* for trend
Patients with age ≥ 40 years	1 (Ref)	2.02 (0.67-6.11) 0.214	3.36 (1.58-7.18) 0.002	4.02 (1.57-10.32) 0.004	< 0.001
Exclude Patients with BMI < 18.5 or ≥ 28.0 kg/m^2^	1 (Ref)	1.88 (0.68-5.22) 0.223	3.75 (1.62-8.69) 0.002	3.86 (1.65-8.99) 0.002	0.001
Exclude CHD patients	1 (Ref)	1.81 (0.67-4.87) 0.240	3.00 (1.23-7.28) 0.015	3.16 (1.29-7.74) 0.012	0.007
Exclude DM patients	1 (Ref)	1.75 (0.36-8.57) 0.491	**3.38 (1.97-8.86) 0.001**	**3.55 (1.08-11.64) 0.036**	**0.025**
Exclude smoker	1 (Ref)	2.06 (0.69-6.10) 0.193	**3.18 (1.19-8.50) 0.021**	**3.47 (1.27-9.46) 0.015**	**0.009**
Exclude drinker	1 (Ref)	2.27 (0.81-6.39) 0.119	**3.58 (1.46-8.83) 0.005**	**3.27 (1.32-8.06) 0.010**	**0.007**
Patients with NYHA class I or II	1 (Ref)	1.61 (0.58-4.44) 0.359	**3.63 (1.57-8.39) 0.003**	**4.07 (1.75-9.47) 0.001**	**< 0.001**
Patients with eGFR ≥ 60 ml/min/1.73m^2^	1 (Ref)	2.42 (0.74-7.92) 0.145	**3.07 (1.39-6.79) 0.006**	**3.71 (1.34-10.26) 0.012**	**0.002**
Patients with LAD < 50 mm	1 (Ref)	1.79 (0.58-5.51) 0.314	**3.30 (1.55-7.02) 0.002**	**3.98 (1.55-10.21) 0.004**	**< 0.001**
Patients with LVEF ≥ 50%	1 (Ref)	1.75 (0.57-5.37) 0.326	**3.23 (1.52-6.87) 0.002**	**3.75 (1.46-9.63) 0.006**	**< 0.001**
Patients with CHA_2_DS_2_-VASc score ≥ 2	1 (Ref)	1.64 (0.64-5.28) 0.558	**2.79 (1.17-6.63) 0.020**	**3.34 (1.01-8.41) 0.048**	**0.017**
Patients with HAS-BLED score < 3	1 (Ref)	2.64 (0.82-8.48) 0.104	**3.13 (1.37-7.14) 0.007**	**4.95 (1.81-13.57) 0.002**	**< 0.001**

HRs were calculated via Cox proportional hazards models. The models were adjusted for age, sex, smoking status, mean SBP, mean DBP, BMI, NYHA classification, CHA_2_DS_2_-VASc score, HAS-BLED score, type of ablation, CHD, DM, WBC, ALB, eGFR, CRP, hsCRP, NT-pro BNP, TG, LAD, and LVEF. Bold values indicate statistical significance. A *P* value < 0.05 indicated a significant difference. Abbreviations: AF, atrial fibrillation; ALB, albumin; BMI, body mass index; BP, blood pressure; CHD, coronary heart disease; CI, confidence interval; CRP, C-reactive protein; DBP, diastolic blood pressure; DM, diabetes mellitus; eGFR, estimated glomerular filtration rate; HR, hazard ratio; hsCRP, high-sensitivity C-reactive protein; LAD, left atrial diameter; LVEF, left ventricular ejection fraction; NLR, neutrophil-to-lymphocyte ratio; NT-pro BNP, N-terminal pro-B-type natriuretic peptide; NYHA, New York Heart Association; SBP, systolic blood pressure; TG, triglyceride; WBC, white blood cell count.

## Abbreviations

ACEI: angiotensin-converting enzyme inhibitor
ACME: average causal mediation effect
ACT: activated clotting time
ADE: average direct effect
AF: atrial fibrillation
ALB: albumin
AP: attributable proportion
ARB: angiotensin II receptor blocker
ARNI: angiotensin receptor-neprilysin inhibitor
AUC: area under the curve
BMI: body mass index
BP: blood pressure
CBA: cryoballoon ablation
CCB: calcium channel blocker
CHD: coronary heart disease
CI: confidence interval
CRP: C-reactive protein
CTA: cardiac computed tomography angiography
CVD: cardiovascular disease
DBP: diastolic blood pressure
DM: diabetes mellitus
eGFR: estimated glomerular filtration rate
HbA1c: glycosylated hemoglobin
HDL-C: high-density lipoprotein cholesterol
HR: hazard ratio
hsCRP: high-sensitivity C-reactive protein
INTM: interaction term
K-M: Kaplan-Meier
LAD: left atrial diameter
LYMPH: lymphocytes count
LVEF: left ventricular ejection fraction
NEUT: neutrophils count
NLR: neutrophil-to-lymphocyte ratio
NT-pro BNP: N-terminal pro-B-type natriuretic peptide
NYHA: New York Heart Association
OR: odds ratio
RCS: restricted cubic spline
RERI: relative excess risk due to interaction
RFA: radiofrequency ablation
ROC: receiver operating characteristic
SBP: systolic blood pressure
SI: synergy index
TEE: transesophageal echocardiography
TG: triglyceride
WBC: white blood cell count
