# Supplementary material for: Neutrophil-to-lymphocyte ratio as a predictor of post-ablation recurrence in hypertensive patients with paroxysmal atrial fibrillation
Source: Int J Med Sci. 2026 Jan 1;23(2):529–42. doi: 10.7150/ijms.118572 (PMC12825140; doi:10.7150/ijms.118572)
Supplement: Supplementary file 1 — Supplementary figures and tables. [file ijmsv23p0529s1.pdf]

Supplementary materials

Neutrophil-to-lymphocyte ratio as a predictor of post-ablation recurrence in hypertensive patients with paroxysmal atrial fibrillation

Zixi Zhang [#], Chao Sun [#], Siyuan Tan, Yichao Xiao, Tao Tu, Qiuzhen Lin, Chan Liu, Chaoshuo Liu, Cancan Wang, Murong Xie *, Qiming Liu *

Supplementary figure legends

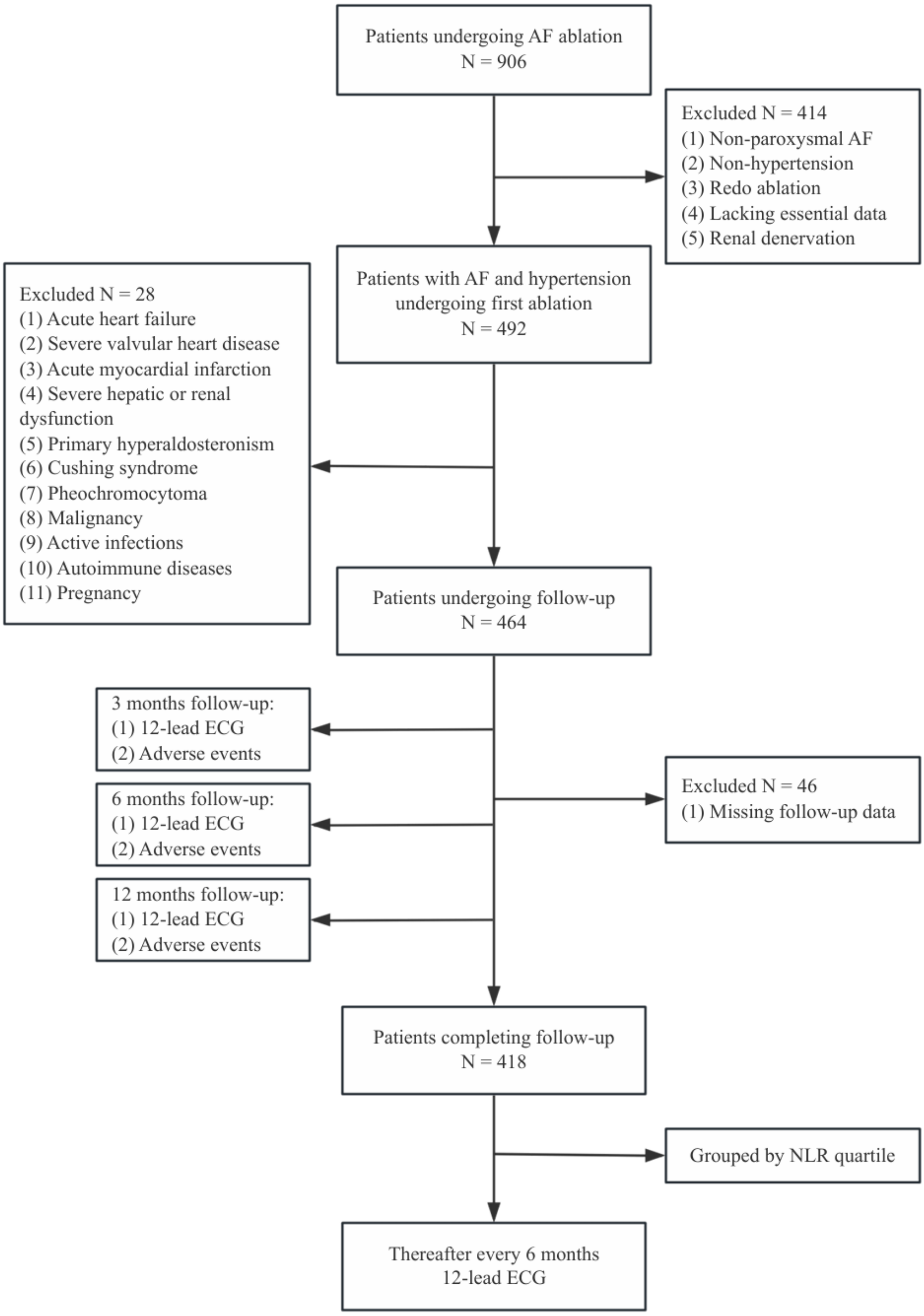

Supplementary Figure 1. Flow diagram for participants included in the study.

Abbreviations: AF, atrial fibrillation; ECG, electrocardiogram; NLR, neutrophil–to–lymphocyte ratio.

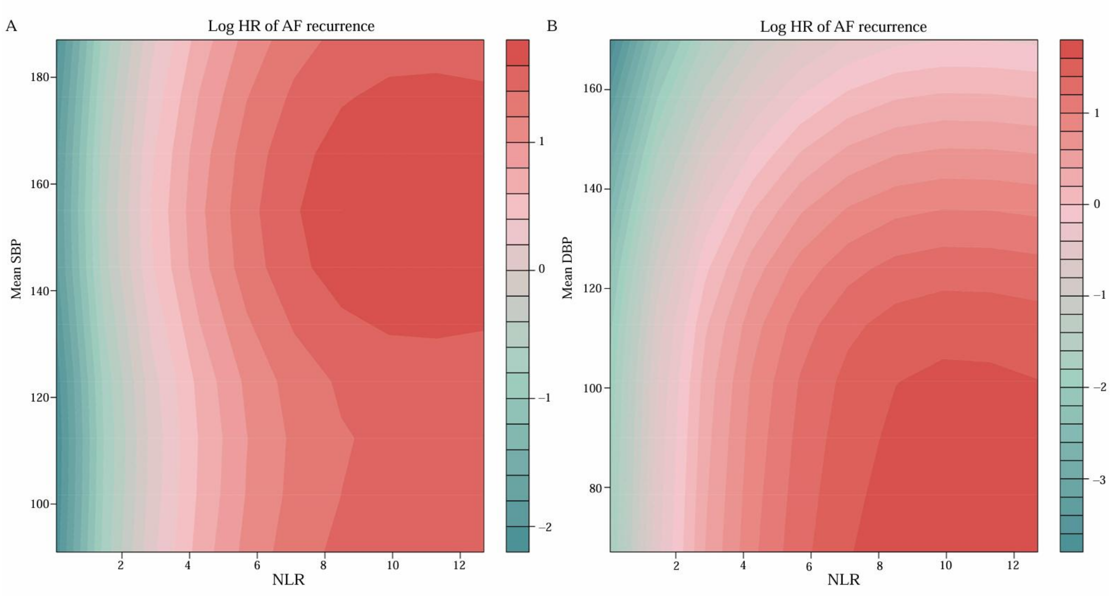

Supplementary Figure 2. Contour plots of the logHR for AF recurrence based on NLR and mean SBP/DBP.

Abbreviations: AF, atrial fibrillation; HR, hazard ratio; DBP, diastolic blood pressure; NLR, neutrophil–to–lymphocyte ratio; SBP, systolic blood pressure.

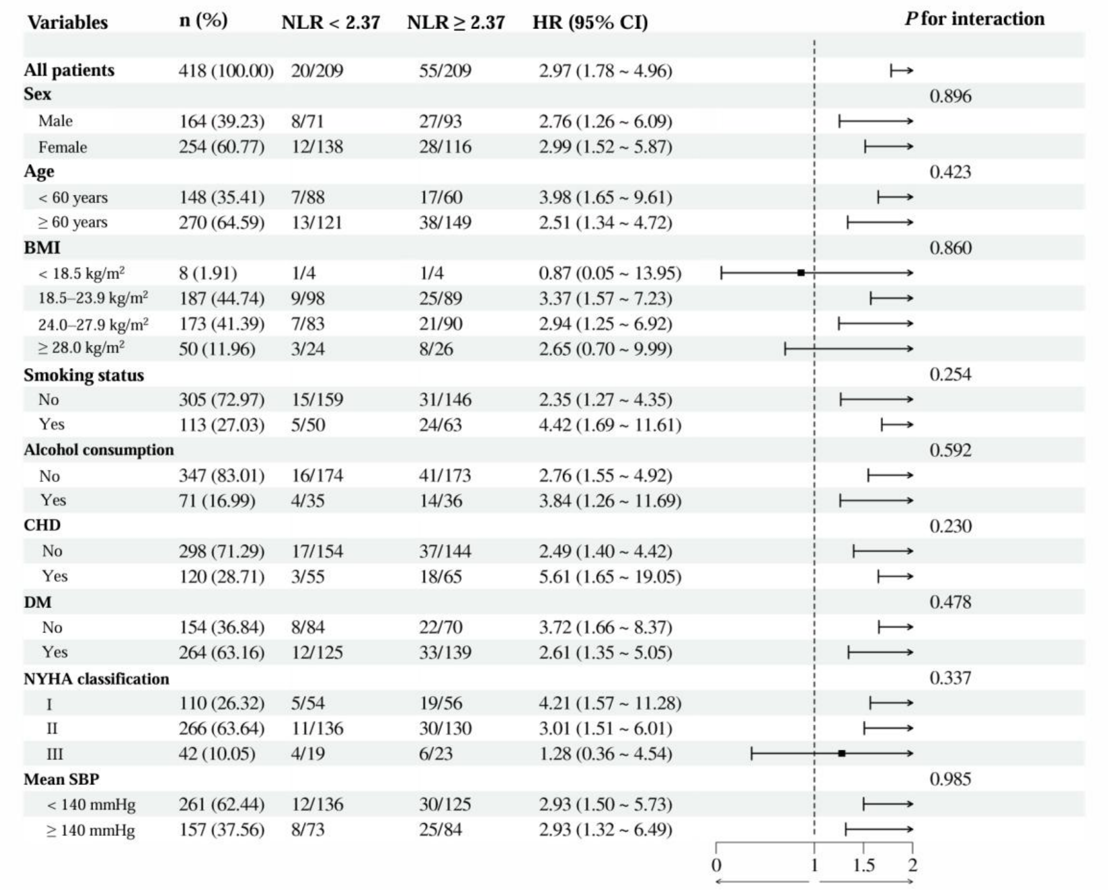

Variables	n (%)	NLR < 2.37	NLR ≥ 2.37	HR (95% CI)	*P* for interaction
**All patients**	418 (100.00)	20/209	55/209	2.97 (1.78 ~ 4.96)	
**Sex**					0.896
Male	164 (39.23)	8/71	27/93	2.76 (1.26 ~ 6.09)	
Female	254 (60.77)	12/138	28/116	2.99 (1.52 ~ 5.87)	
**Age**					0.423
< 60 years	148 (35.41)	7/88	17/60	3.98 (1.65 ~ 9.61)	
≥ 60 years	270 (64.59)	13/121	38/149	2.51 (1.34 ~ 4.72)	
**BMI**					0.860
< 18.5 kg/m²	8 (1.91)	1/4	1/4	0.87 (0.05 ~ 13.95)	
18.5–23.9 kg/m²	187 (44.74)	9/98	25/89	3.37 (1.57 ~ 7.23)	
24.0–27.9 kg/m²	173 (41.39)	7/83	21/90	2.94 (1.25 ~ 6.92)	
≥ 28.0 kg/m²	50 (11.96)	3/24	8/26	2.65 (0.70 ~ 9.99)	
**Smoking status**					0.254
No	305 (72.97)	15/159	31/146	2.35 (1.27 ~ 4.35)	
Yes	113 (27.03)	5/50	24/63	4.42 (1.69 ~ 11.61)	
**Alcohol consumption**					0.592
No	347 (83.01)	16/174	41/173	2.76 (1.55 ~ 4.92)	
Yes	71 (16.99)	4/35	14/36	3.84 (1.26 ~ 11.69)	
**CHD**					0.230
No	298 (71.29)	17/154	37/144	2.49 (1.40 ~ 4.42)	
Yes	120 (28.71)	3/55	18/65	5.61 (1.65 ~ 19.05)	
**DM**					0.478
No	154 (36.84)	8/84	22/70	3.72 (1.66 ~ 8.37)	
Yes	264 (63.16)	12/125	33/139	2.61 (1.35 ~ 5.05)	
**NYHA classification**					0.337
I	110 (26.32)	5/54	19/56	4.21 (1.57 ~ 11.28)	
II	266 (63.64)	11/136	30/130	3.01 (1.51 ~ 6.01)	
III	42 (10.05)	4/19	6/23	1.28 (0.36 ~ 4.54)	
**Mean SBP**					0.985
< 140 mmHg	261 (62.44)	12/136	30/125	2.93 (1.50 ~ 5.73)	
≥ 140 mmHg	157 (37.56)	8/73	25/84	2.93 (1.32 ~ 6.49)	

Supplementary Figure 3. Forest plot depicting subgroup analyses of the associations between NLR and AF recurrence.

$P < 0.05$ indicates statistical significance.

Abbreviations: AF, atrial fibrillation; BMI, body mass index; CHD, coronary heart disease; CI, confidence interval; DM, diabetes mellitus; HR, hazard ratio; NLR, neutrophil–to–lymphocyte ratio; NYHA, New York Heart Association; SBP, systolic blood pressure.

Supplementary Table 1. Univariate Cox regression analysis of NLR and AF recurrence.

**Variables**	**AF recurrence**	
	HR (95% CI)	*P* value
Age	1.01 (0.98–1.04)	0.475
**Sex**		
Male	1 (Reference)	
Female	0.72 (0.46–1.13)	0.152
**Alcohol consumption**		
No	1 (Reference)	
Yes	1.65 (0.97–2.80)	0.065
**Smoking status**		
No	1 (Reference)	
Yes	1.85 (1.16–2.95)	**0.009**
Mean SBP	1.00 (0.99–1.02)	0.439
Mean DBP	0.99 (0.97–1.01)	0.199
BMI	1.01 (0.94–1.09)	0.839
**NYHA classification**		
I	1 (Reference)	
II	0.67 (0.41–1.11)	0.120
III	1.08 (0.52–2.26)	0.837
CHA_2DS_2-VASc score	0.93 (0.81–1.06)	0.278
HAS-BLED score	1.04 (0.84–1.29)	0.735
CHD	0.98 (0.59–1.62)	0.925
DM	0.85 (0.53–1.35)	0.487
ACEI/ARB	0.88 (0.56–1.40)	0.592
ARNI	1.12 (0.68–1.83)	0.658
β-blockers	0.87 (0.50–1.51)	0.625
CCBs	1.02 (0.60–1.71)	0.955
Diuretics	1.16 (0.65–2.07)	0.622
WBC	1.13 (1.01–1.27)	**0.003**
Hb	1.01 (1.00–1.02)	0.173
RBC	1.18 (0.82–1.69)	0.380
PLT	1.00 (1.00–1.01)	0.550
ALB	0.99 (0.93–1.06)	0.793
eGFR	0.99 (0.98–1.00)	0.108
CRP	1.01 (0.99–1.03)	0.261
hsCRP	1.01 (0.95–1.08)	0.686
NT-pro BNP	1.00 (1.00–1.00)	0.491
FPG	1.01 (0.83–1.24)	0.885
HbA1c	1.09 (0.85–1.40)	0.515
TG	1.18 (1.02–1.38)	**0.030**
TC	1.05 (0.83–1.34)	0.690

HDL-C	0.54 (0.24–1.25)	0.153
LDL-C	0.98 (0.74–1.31)	0.917
LAD	1.00 (0.96–1.05)	0.982
LVDd	1.02 (0.97–1.08)	0.395
LVEF	1.01 (0.97–1.05)	0.705
**Type of ablation**		
RFA	1 (Reference)	
CBA	1.01 (0.63–1.62)	0.970

Bold values indicate statistical significance. A *P* value < 0.05 indicated a significant difference.

Abbreviations: ACEI, angiotensin-converting enzyme inhibitor; AF, atrial fibrillation; ALB, albumin; ARB, angiotensin II receptor blocker; ARNI, angiotensin receptor-neprilysin inhibitor; BMI, body mass index; CBA, cryoballoon ablation; CCB, calcium channel blocker; CHD, coronary heart disease; CRP, C-reactive protein; DBP, diastolic blood pressure; DM, diabetes mellitus; eGFR, estimated glomerular filtration rate; FPG, fasting plasma glucose; Hb, hemoglobin; HbA1c, glycosylated hemoglobin; HDL-C, high-density lipoprotein cholesterol; hsCRP, high-sensitivity C-reactive protein; LAD, left atrial diameter; LDL-C, low-density lipoprotein cholesterol; LVDd, left ventricular end diastolic diameter; LVEF, left ventricular ejection fraction; NLR, neutrophil–to–lymphocyte ratio; NT-pro BNP, N-terminal pro-B-type natriuretic peptide; NYHA, New York Heart Association; PLT, platelet; RBC, red blood cell count; RFA, radiofrequency ablation; SBP, systolic blood pressure; TC, total cholesterol; TG, triglyceride; WBC, white blood cell count.

Supplementary Table 2. VIFs for covariates in Cox regression models of NLR and AF recurrence.

Variables	VIF for AF recurrence
Age	1.55
Sex	1.40
Smoking status	1.25
Mean SBP	1.61
Mean DBP	1.63
BMI	1.23
NYHA classification	1.20
CHA_2DS_2-VASc score	2.50
HAS-BLED score	2.35
CHD	1.21
DM	1.35
WBC	1.10
ALB	1.12
eGFR	1.33
CRP	1.08
hsCRP	1.07
NT-pro BNP	1.32
TG	1.07
LAD	1.63
LVEF	1.31
Type of ablation	1.10

Abbreviations: AF, atrial fibrillation; ALB, albumin; BMI, body mass index; CHD, coronary heart disease; CRP, C-reactive protein; DBP, diastolic blood pressure; DM, diabetes mellitus; eGFR, estimated glomerular filtration rate; hsCRP, high-sensitivity C-reactive protein; LAD, left atrial diameter; LVEF, left ventricular ejection fraction; NLR, neutrophil–to–lymphocyte ratio; NT-pro BNP, N-terminal pro-B-type natriuretic peptide; NYHA, New York Heart Association; SBP, systolic blood pressure; TG, triglyceride; VIF, variance inflation factor; WBC, white blood cell count.

Supplementary Table 3. Diagnostic performance evaluation metrics for identifying AF recurrence via inflammatory indices.

Index	AUC (95% CI)	*P* value	Cutoff	Sensitivity (95% CI)	Specificity (95% CI)	PPV (95% CI)	NPV (95% CI)
NLR	0.69 (0.63–0.76)	1(Ref)	2.61	0.65 (0.60–0.70)	0.69 (0.59–0.80)	0.91 (0.87–0.94)	0.30 (0.23–0.37)
CRP	0.51 (0.43–0.59)	0.001	2.63	0.66 (0.61–0.71)	0.45 (0.34–0.57)	0.85 (0.80–0.89)	0.23 (0.16–0.29)
hsCRP	0.54 (0.47–0.62)	0.003	1.18	0.68 (0.63–0.73)	0.45 (0.34–0.57)	0.85 (0.81–0.89)	0.24 (0.17–0.31)

A *P* value < 0.05 indicated a significant difference.

Abbreviations: AF, atrial fibrillation; AUC, area under the curve; CI, confidence interval; CRP, C-reactive protein; hsCRP, high-sensitivity C-reactive protein; NLR, neutrophil–to–lymphocyte ratio; NPV, negative predictive value; PPV, positive predictive value.

Supplementary Table 4. Mediating relationship between the NLR and AF recurrence according to demographic, metabolic, and inflammatory marker data.

Mediators	ADE		ACME		Proportion mediated (%)
	Coefficients (95% CIs)	*P* value	Coefficients (95% CIs)	*P* value	
Age	0.02949 (0.02139–0.03000)	< **0.001**	0.00004 (–0.00148–0)	0.880	0.00119
BMI	0.02960 (0.02159–0.03000)	< **0.001**	–0.00009 (–0.00200–0)	0.800	–0.00300
Mean SBP	0.02961 (0.02144–0.04000)	< **0.001**	0.00009 (–0.00193–0)	0.680	0.00315
Mean DBP	0.02948 (0.02143–0.04000)	< **0.001**	–0.00003 (–0.00243–0)	0.960	–0.00112
eGFR	0.02879 (0.02036–0.03000)	< **0.001**	0.00091 (–0.00009–0)	0.160	0.03051
ALB	0.02948 (0.02138–0.03000)	< **0.001**	0.00004 (–0.00162–0)	0.840	0.00141
NT-pro BNP	0.02962 (0.02148–0.03000)	< **0.001**	0.00004 (–0.00124–0)	0.920	0.00129
CRP	0.02925 (0.02003–0.04000)	< **0.001**	0.00038 (–0.00633–0)	0.960	0.01289
hsCRP	0.02957 (0.02119–0.03000)	< **0.001**	–0.00005 (–0.00058–0)	0.800	–0.00182
FPG	0.02948 (0.02128–0.04000)	< **0.001**	0.00001 (–0.00102–0)	0.920	0.00043
HbA1c	0.02951 (0.02140–0.04000)	< **0.001**	0.00003 (–0.00111–0)	0.840	0.00099
TG	0.02876 (0.02085–0.03000)	< **0.001**	0.00124 (–0.00039–0)	0.200	0.04142
TC	0.02972 (0.02234–0.03000)	< **0.001**	–0.00015 (–0.00091–0)	0.920	–0.00514
LAD	0.02971 (0.02178–0.03000)	< **0.001**	–0.00032 (–0.00176–0)	0.600	–0.01099
LVEF	0.02951 (0.02140–0.03000)	< **0.001**	0.00002 (–0.00093–0)	0.720	0.00061

Adjusted for age, sex, smoking status, mean SBP, mean DBP, BMI, NYHA classification, CHA_2DS_2-VASc score, HAS-BLED score, type of ablation, CHD, DM, WBC, ALB, eGFR, CRP, hsCRP, NT-pro BNP, TG, LAD, and LVEF. Bold values indicate statistical significance. A *P* value

< 0.05 indicated a significant difference.

Abbreviations: ACME, average causal mediation effect; ADE, average direct effect; AF, atrial fibrillation; ALB, albumin; BMI, body mass index; CHD, coronary heart disease; CI, confidence interval; CRP, C-reactive protein; DBP, diastolic blood pressure; DM, diabetes mellitus; eGFR, estimated glomerular filtration rate; hsCRP, high-sensitivity C-reactive protein; LAD, left atrial diameter; LVEF, left ventricular ejection fraction; NLR, neutrophil–to–lymphocyte ratio; NT-pro BNP, N-terminal pro-B-type natriuretic peptide; NYHA, New York Heart Association; SBP, systolic blood pressure; TG, triglyceride; WBC, white blood cell count.
